# IgG antibody titers to SARS-CoV-2 reveal a distinct efficacy of multiple sclerosis-modifying therapies to curtail lymphocyte compartments

**DOI:** 10.1177/17562864211022109

**Published:** 2021-06-03

**Authors:** Jürgen Wienands

**Affiliations:** Institute of Cellular and Molecular Immunology, University Medical Center Göttingen, George August University of Göttingen, Humboldtallee 34, Göttingen, 37073, Germany

**Keywords:** vaccination, Covid-19, humoral immune response, antibody production, multiple sclerosis

The adaptive immune system is composed of interacting B- and T-lymphocyte compartments with different roles in host defense and autoimmune pathogenesis. Pharmaceutical intervention with the latter has been shown to be highly beneficial to patients with multiple sclerosis (MS). However, these treatments come at the expense of a possibly reduced capacity to cope with invasive pathogens and the likelihood of impaired responses to vaccinations.^1^ The current COVID-19 pandemic and the sometimes controversial discussion on vaccination strategies underscore the medical need for a more detailed assessment of how exactly and to what extent disease-modifying therapies (DMTs) compromise physiological immune capabilities. Such an inventory paves the way for evidence-based recommendations of balanced treatment options. Towards that goal, Achiron *et al.*^2^ measured IgG antibody responses and absolute numbers of lymphocytes in cohorts of DMT-treated patients with MS who were vaccinated against severe acute respiratory syndrome coronavirus 2 (SARS-CoV-2). The results obtained were compared with sex- and age-matched control cohorts of healthy vaccinees or patients without medication. The impact of three routinely applied DMTs that effectively modulate the survival or functionality of B cells and T cells were individually assessed: (a) cladribine, a purine analog with genotoxicity to both lymphocyte lineages;^3^ (b) fingolimod that antagonizes sphingosine-1-phosphate (SP1) action and thereby inhibits the migration of lymphocytes to and out of secondary immune organs;^4^ (c) the humanized monoclonal antibody ocrelizumab that effectively depletes peripheral B lymphocytes upon its binding to the B-lineage surface marker CD20 and subsequently activates various immune effector functions.^5^ These DMTs showed striking differences in modulating the humoral immune response to the mRNA-based COVID-19 vaccine BNT162b2, which is based on a modified mRNA encoding the viral spike protein.^6^

A first and remarkable message from this study is that the BNT162b2 vaccine elicited similar titers of SARS-CoV-2-specific serum IgG in healthy individuals and untreated patients with MS. Likewise, vaccination with BNT162b2 around 4 months after the last cladribine treatment dose induced normal IgG serum titers against the viral spike glycoprotein. Even though the absolute lymphocyte counts were still reduced, it is likely that this response provided a large degree of protection against COVID-19 symptoms because the ELISA quantification assay used in this study had been previously matched with the quality of the IgG antibodies in terms of their antigen affinity and their general neutralization capacity. However, and in marked contrast, neither fingolimod- nor ocrelizumab-treated patients with MS showed robust IgG antibody responses to the SARS-CoV-2 spike protein even when vaccinated 6.4 months after the last medication with ocrelizumab. Lymphocyte counts were more frequently and more substantially reduced. Remaining lymphocytes in the ocrelizumab-treated cohort were not further classified but represented most likely T cells that, with the exception of a minor CD20-positive subpopulation, are not affected by this depleting antibody. The study raises three obvious questions. Firstly, can we explain the data at a cellular and molecular level? Secondly, what are their implications for vaccination recommendations to DMT-treated patients, and finally, is the DMT-influenced level of protection against more serious COVID-19 disease symptoms also sufficient to prevent the production of contagious viral particles?

The presented data are consistent with our mechanistic understanding of humoral immunity (from the Greek immunity ‘associated with body fluids’) as well as with our current knowledge about the mode of action of the applied DMTs.^3–5^ It needs to be mentioned here that full protection against a previously encountered viral or bacterial pathogen or any kind of ‘foreign’ antigen, including toxins, critically requires the production of so-called neutralizing antibodies, most notably of the IgG class of Ig isotypes.^7^ Neutralizing antibodies usually bind their cognate antigen with high affinity and ‘inactivate’ the pathogen by various immune effector mechanisms before it can impart any harm to the organism that, for example, results from virus entry into host cells and their subsequent destruction. Soluble IgG in the serum or other body fluids is produced by plasma cells that represent terminally differentiated B cells. In fact, Ig-secreting, long-lived plasma cells substantially contribute to humoral immunity. The second and a more indirect source of neutralizing IgG antibodies is provided by memory B cells, which, unlike plasma cells do not secrete Ig molecules but instead express membrane-bound Ig molecules on their surface as part of a signaling-competent, B-cell antigen receptor.^8^ However, the very low activation threshold of IgG-positive memory B cells facilitates their instant differentiation into IgG-secreting plasma cells upon antigen recall. These ‘ready-to-go’ B cells as well as their antigen-unexperienced IgM-positive precursors are all CD20-positive and therefore susceptible to ocrelizumab-mediated depletion. The resulting loss of non-switched and class-switched B cells prevents the production of plasma cells and explains why IgG antibody titers are neither induced nor maintained even though plasma cells themselves do not express the CD20 marker and hence, remain unaffected by ocrelizumab. Fingolimod interferes with proper antibody responses in many ways.^4^ Most prominently studied is the failure of lymphocytes to migrate along SP1 gradients in the circulation, which is a consequence of fingolimod-mediated desensitization of their SP1 receptor but is a requisite for the correct navigation and niche confinement of T cells and B cells prior to and during immune responses. The disturbed lymphocyte homing and trafficking processes directly compromise the activation of naïve and memory B cells but also impinge on T-cell helper functions that are of particular importance for mounting a primary antibody response to protein antigens. In accordance with these mechanisms, Achiron *et al.*^2^ found little to no SARS-CoV-2-specific IgG antibodies associated with very low absolute numbers of peripheral lymphocytes. Similar to fingolimod, cladribine impacts on both lymphocyte lineages.^3^ However, it appears to deplete different subpopulations with distinct efficacy depending on the expression levels of deoxycytidine kinase that activates the cladribine pro-drug by phosphorylation. Memory B cells rather than naïve B cells or plasma cells seem to be preferentially susceptible to cladribine treatment. Similarly, the memory T-cell compartment and CD4-positive T-helper cells show long-term depletion kinetics, while CD8-positive cytotoxic T cells have been reported to be more moderately affected. The exact predilection of cladribine for individual immune cell (sub-) populations is still under investigation but the available data including those presented by Achiron *et al.*^2^ are compatible with a remaining lymphocyte repertoire that suffices to mount almost normal IgG antibody responses.

Altogether the results and current concepts provide a first basis for a refinement of vaccination protocols for patients with MS. In the absence of DMTs, vaccination of patients with MS will elicit quantitatively and probably also qualitatively normal serum titers of anti-SARS-CoV-2 antibodies. The adverse side effects were reported to be similar to those observed in healthy vaccinees.^9^ Fingolimod or ocrelizumab-treated patients with MS lacked robust antibody responses. However, the postvaccination status of their cellular immunity that is orchestrated by T cells and natural killer cells remains to be elucidated in detail. Given the expression profile of CD20, ocrelizumab treatment will allow for substantial induction of CD4- as well as CD8-positive memory T-cell compartments that can efficiently attack SARS-CoV-2-infected cells and hence mitigate the course of COVID-19.^10^ The vaccinees nonetheless may still be susceptible to SARS-CoV-2 infection because they lack neutralizing antibodies and may, therefore, produce and spread contagious viral particles to some extent. As with ocrelizumab-treated patients with MS, there appears to be no obvious objection to COVID-19 vaccination of cladribine-treated individuals. However, the question here is how long lasting will be the humoral and cellular immunity. However, this uncertainty also applies to healthy vaccinees and is one of the many aspects needed to be clarified in order to get back to normal life.
